# Prevalence and correlates of achieving recommended physical activity levels among children living in rural South Asia—A multi-centre study

**DOI:** 10.1186/s12889-016-3353-x

**Published:** 2016-08-02

**Authors:** Krithiga Shridhar, Christopher Millett, Anthony A. Laverty, Dewan Alam, Amit Dias, Joseph Williams, Preet K. Dhillon

**Affiliations:** 1 Centre for Chronic Conditions and Injuries, Public Health Foundation of India, 4th Floor, Plot.No.47, Sector 44, Gurgaon, 122002 Haryana India; 2 Department of Primary Care and Public Health, Imperial College, Reynolds Building, Charing Cross Campus, London, UK; 3 Centre for Global Health Research, Li KaShing Knowledge Institute, St. Michael’s Hospital, Toronto, Canada; 4 Goa Medical College, Sangath, Bardez, Goa India; 5 Voluntary Health Services, Chennai, India

**Keywords:** Physical activity, Children, South Asia, LMIC, Active travel, Sedentary activity, BMI-z- score, Anthropometrics, Waist- z- score

## Abstract

**Background:**

We report the prevalence of recommended physical activity levels (RPALs) and examine the correlates of achieving RPALs in rural South Asian children and analyse its association with anthropometric outcomes.

**Methods:**

This analysis on rural South Asian children aged 5–14 years (*n* = 564) is a part of the Chronic Disease Risk Factor study conducted at three sites in India (Chennai *n* = 146; Goa *n* = 218) and Bangladesh (Matlab; *n* = 200). Data on socio-demographic and lifestyle factors (physical activity (PA); diet) were collected using an interviewer-administered questionnaires, along with objective anthropometric measurements. Multivariate logistic regression models were used to examine whether RPALs (active travel to school (yes/no); leisure-time PA ≥ 1 h/day; sedentary-activity ≤ 2 h/day) were associated with socio-demographic factors, diet and other forms of PA. Multivariate linear regression models were used to investigate associations between RPALs and anthropometrics (BMI- and waist z-scores).

**Results:**

The majority of children (71.8 %) belonged to households where a parent had at least a secondary education. Two-thirds (66.7 %) actively travelled to school; 74.6 % reported ≥1 h/day of leisure-time PA and 55.7 % had ≤2 h/day of sedentary-activity; 25.2 % of children reported RPALs in all three dimensions. Older (10–14 years, OR = 2.0; 95 % CI: 1.3, 3.0) and female (OR = 1.7; 95 % CI: 1.1, 2.5) children were more likely to travel actively to school. Leisure-time PA ≥ 1 h/day was more common among boys (OR = 2.5; 95 % CI: 1.5, 4.0), children in Matlab, Bangladesh (OR = 3.0; 95 % CI: 1.6, 5.5), and those with higher processed-food consumption (OR = 2.3; 95 % CI: 1.2, 4.1). Sedentary activity ≤ 2 h/day was associated with younger children (5–9 years, OR = 1.6; 95 % CI: 1.1, 2.4), children of Goa (OR = 3.5; 95 % CI: 2.1, 6.1) and Chennai (OR = 2.5; 95 % CI: 1.5, 4.3) and low household education (OR = 2.1; 95 % CI: 1.1, 4.1). In multivariate analyses, sedentary activity ≤ 2 h/day was associated with lower BMI-z-scores (β = −0.3; 95 % CI: −0.5, −0.08) and lower waist-z-scores (β = −1.1; 95 % CI: −2.2, −0.07).

**Conclusion:**

Only one quarter of children in these rural areas achieved RPAL in active travel, leisure and sedentary activity. Improved understanding of RPAL in rural South Asian children is important due to rapid socio-economic transition.

## Background

Physical activity in children improves cardio-respiratory, muscular, bone and mental health [1–4]. It decreases the likelihood of overweight and obesity and the development of non-communicable diseases (NCDs) in childhood and adulthood [1–4]. Some programming of adult-onset NCDs can take place during childhood [1, 5] and are preventable through early life-style modifications [6]. Physical activity is an established modifiable risk exposure, for which earlier tracking and intervention will help in attaining a beneficial effect of physical activity patterns in adult life [1]. The World Health Organization (WHO) recommends 1 h of moderate to vigorous physical activity for children everyday [4]. Sedentary activity less than 2 h per day is beneficial for physical, social and academic performance of children [1–3, 7]. ‘Active travel to school’ such as walking and cycling (versus use of motorized transport) improves cardiovascular fitness and thus is recommended as well [8].

Although data in children are limited in South Asia [9], physical activity patterns and the risks associated with various forms of physical inactivity (motorized transport to school, long TV viewing, no outdoor games etc.) have been reported in children and adolescents in some urban settings [1, 3, 9–19]. Across the various regions of urban South Asia a high prevalence of physical inactivity (18.3 to 67.4 %) have been reported among children [1, 9]. Transition in physical activity patterns (i.e., from being active to more sedentary) among children is also an increasing concern globally [20, 21]. Data from rural South Asia are sparse [9], partly because physical inactivity or sedentary behaviour is perceived to be a problem of urban environments and among more affluent communities. India and Bangladesh are low-and-middle income countries (LMICs) where nearly three-fourths of children aged between 5 and 14 years live in rural areas [22, 23]. NCD risk factors, [24–26] and sedentary behaviour are frequently reported in rural adult populations, [27] yet, there are no strategic guidelines for physical activity in children in these regions and physical activity data in children from rural areas will help inform policy decisions [28, 29].

In this study, we report the prevalence and correlates of recommended physical activity levels (RPALs) through leisure-time physical activity (LTPA) and active travel alongside sedentary activity from three different rural regions of India (Chennai and Goa) and Bangladesh (Matlab). We examine associations between different forms of physical activity and measures of adiposity.

## Methods

### Study population

Study participants were part of the Chronic Disease Risk Factor (CDRF) study. The CDRF is a multi-centre, cross-sectional study of NCD risk factors and outcomes among rural populations in South Asia [30, 31]. The survey was conducted by the South Asia Network for Chronic Diseases (SANCD), Public Health Foundation of India in partnership with local organizations at the study sites: Sangath in Goa, Voluntary Health Services in Chennai, and the International Centre for Diarrheal Disease Research, Matlab in Bangladesh. At each site, sampling frames that reflected local demographics and access to healthcare were constructed. Each site was selected on the basis of being a typical rural community in each setting, reflecting the local demographics and having access to healthcare facilities to support the collection of physical measurements. At each site, households in consecutive village sections, from the healthcare centre outwards, were sampled until households numbered at least 250 and terminated at the end of a street, block or cluster of homes within a village (*n* = 308 households in Matlab, 309 in Goa, 257 in Chennai). All household residents of 2+ years resident in the area for at least 6 months of the year were invited to participate (*n* = 1143 in Matlab, 1212 in Goa, 940 in Chennai). Response rates were 92.9 % in Goa, 96.1 % in rural Chennai and 96.1 % in Matlab. This analysis included only children aged 5 to 14 years who provided informed consent to participate in the CDRF project. A total of *n* = 564 children aged 5 to 14 years were enrolled in the study from rural regions of India (*n* = 146 from Chennai; *n* = 218 from Goa) and Bangladesh (*n* = 200 from Matlab). An urban population was also sampled in Chennai, but excluded from our analysis as numbers (*n* = 76) were insufficient for urban–rural comparisons.

### Data collection

Data were collected from November, 2011 to March, 2013 in Chennai, October, 2011 to March, 2013 in Goa and June, 2011 to May, 2012 in Matlab. Each consenting participant received a structured, interviewer-administered questionnaires, which was translated and back translated to the local language of the study site (Tamil in Chennai; Konkani in Goa; Bengali in Bangladesh). Household-level data were collected on non-health expenditures, utilities, health insurance, exposure to indoor biomass fuel and monthly consumption of sugar, salt and oil. Individual-level data were captured by directly administering the questionnaires to children from 10 to 14 years and to the mother or other care-taker if the child was 2 to 9 years of age. Details on personal factors (e.g., siblings, order of birth, health status, quality of life), medical history, use of health care services, lifestyle habits (e.g., diet, physical activity), and outcomes (e.g., disability) were collected. Physical measurements such as anthropometrics (e.g., height, weight, waist, hip and arm circumferences) were collected by trained personnel. Socio-economic status (SES) for the household was calculated based on tertiles from total monthly expenditures for Indian sites and total household asset score for Matlab, Bangladesh. The highest household education status was based on the highest educational level reported among all family members living in the household. Missing data varied between 1 % (e.g., household education status) to 11.5 % (e.g., SES) for different variables.

### Outcome variables

Physical activity measurements in children were adapted from the ‘Indian Migration Study’ Physical Activity Questionnaire (IMS-PAQ) which was validated in adults aged 18+ years across 18 different Indian states [32]. It includes questions about mode of transport to school (e.g., motorized vehicle (car/bus/auto/two-wheeler), bicycle or walk), leisure-time physical activities (e.g., outdoor games/sports both at school and apart from school such as walking, badminton, jogging, cricket) and sedentary activities involving sitting, apart from at school (e.g., reading, watching television, listening to radio, carom, computer/video games). Details about the physical activity in terms of duration per activity (in minutes) and frequency (number of times per day/week/month) were also collected. We were able to capture the same and additional information than World Health Organisation’s Global School-based Health Survey [33] that is culturally appropriate for Indian settings.

Binary variables for mode of travel to school (yes/no any reported mode of active travel), leisure- time physical activity (LTPA) at home and at school, and sedentary activity outside school hours were created. The cut-off level for leisure-time physical activity was based on WHO recommended levels of physical activity for children aged 5 to17 years (i.e., ≥1 h everyday) [4]. The cut-off level for sedentary activity was based on a systematic review of sedentary behavior and health indicators in school-aged children and youth (5–17 years) i.e., ≤ 2 h daily [7].

Secondary outcome variables were anthropometric measurements of children taken by trained personnel. The measurements included two of standing height (to the nearest 1 mm, using a portable stadiometer (Leicester)), two of weight (to the nearest 100 g, using portable digital scales (Tanita), with the participants in light clothing and no shoes), two of each of waist and hip circumference (to the nearest 1 mm, using a taut plastic tape). BMI-z scores are calculated for WHO-reference values using STATA-Macros for children 5 years of age and WHO2007 for children 6–14 years of age which represents external standardization of the study with the WHO reference population [34]. Waist z-scores are residuals adjusted for age and sex which represent internal standardization of the study population. The outcome variables were continuous and adjusted for age and sex.

### Statistical analysis

A standard descriptive summary was expressed as percentages or as the mean and standard deviation (SD) and comparisons between sites made using chi-square or ANOVA tests. Logistic regression models were used to examine socio-demographic and dietary correlates of different types of physical activity/inactivity that are part of RPALs (mode of transport to school, leisure-time physical activity and sedentary activity). Linear regression models were used to examine associations between physical activity status (mode of transport to school, leisure-time physical activity and sedentary activity) and two measures of adiposity: BMI-z and waist-z scores. Models were adjusted for site, SES, highest level of education in the household, processed food consumption, fruit and vegetable consumption and other types of physical activity. All analyses were conducted using STATA version 10 (StataCorp, USA).

## Results

### Summary of participant characteristics

A total of *n* = 564 children aged 5 to 14 years from rural populations of India and Bangladesh were included in our analysis: 146 from Chennai, 218 from Goa and 200 from Matlab. A summary of their socio-demographic characteristics, physical activity patterns, diet and weight-related outcomes are presented in Table 1. Nearly half (48.4 %) were girls. Rural children in Chennai were more likely to live in a household with higher education: 77.6, 73.6 and 60 % of children lived in a household with at least one member educated to secondary school level (Chennai, Goa and Matlab, respectively). Two-thirds of children (66.7 %) actively travelled to school across all sites (69.2 % Chennai, India; 69.4 in Goa, India and 62 % in Matlab, Bangladesh). The majority of children were involved in leisure- time physical activity for ≥ 1 h every day, with the highest prevalence rates (80.2 %) reported in Matlab (59.8 and 78.8 % for Chennai and Goa). Children in Matlab, Bangladesh were much less likely to have ≤ 2 h sedentary activity per day (37 %) compared to children in Chennai (64.8 %) and Goa (67.3 %). Fruit and vegetable consumption was low across all sites (87.8 % consumed <5servings/day). The consumption of processed food (≥1 serving per day) was higher in Matlab and Chennai (44.0 % and 34.9 % respectively, *p* < 0.0001). While Goan children recorded the highest waist-z-scores (mean (SD) 1.3(6.3), *p* < 0.0001), BMI-z-scores were similar across sites (mean (SD) -1.3(1.1)).

**Table 1:** Socio-demographic, physical activity, diet and anthropometric profile of rural South Asian children aged 5–14 years

Mean(SD)/%	Chennai *n* = 146 (25.9 %)	Goa *n* = 218 (38.6 %)	Matlab *n* = 200 (35.5 %)	*p*-value^a^	Total *n* = 564
Age group (years)					
5-9 years	42.4	46.3	45.0	0.7	44.8
10–14 years	57.5	53.6	55.0		55.1
Sex					
% Boys	52.7	56.8	45.0	0.05	51.6
Highest household education status					
0–5 years of education	22.4	26.4	34.0		28.1
6 to 12 years of education	66.4	61.1	51.5	<0.0001	59.0
Graduate/Post graduate	11.2	12.5	14.5		12.8
Socio-economic status^b^(SES)					
Low	45.9	11.2	34.9		28.7
Medium	41.6	29.1	36.1	<0.0001	34.9
High	12.4	59.7	28.9		36.5
Mode of transport to school					
Active transport (walk/bicycle)	69.2	69.4	62.0		66.7
Motorized transport (car, bus, auto, two-wheeler)	30.8	30.6	38.0	0.1	33.3
Leisure-time physical activity					
< 1 h/day	40.1	21.2	19.8		25.4
1–2 h/day	52.2	46.9	43.1		46.8
2.1–4 h/day	6.8	29.2	31.9	<0.0001	24.6
4.1–6 h/day	0.7	1.5	5.0		2.6
> 6 h/day	0.0	1.0	0.0		0.3
Sedentary activity					
< 1 h/day	17.2	19.2	11.5		15.9
1–2 h/day	47.5	48.0	25.5	<0.0001	39.7
2.1–4 h/day	33.7	29.8	43.0		35.6
4.1–6 h/day	0.6	2.8	17.0		7.4
> 6 h/day	0.6	0.0	3.0		1.2
Fruit vegetable intake					
< 5servings/day	95.7	94.5	75.0	<0.0001	87.8
≥ 5servings/day	4.3	5.5	25.0		12.2
Processed food intake					
< 1serving/day	65.1	89.9	56.0	<0.0001	71.5
≥ 1servings/day	34.9	10.1	44.0		28.5
Crude waist circumference (cm)	53.4(7.0)	56.1(7.6)	54.6(5.4)	0.001	54.8(6.8)
Waist-z- scores	−1.4(5.1)	1.3(6.3)	−0.3 (4.3)	<0.0001	0.03(5.4)
Crude BMI	14.7(2.1)	14.9(2.4)	14.7(1.7)	0.40	14.8(2.1)
BMI- z- scores	−1.4(1.1)	−1.3(1.3)	−1.3(0.9)	0.40	−1.3(1.1)

^a^Test of significance for the difference among sites by chi-square test for categorical data and ANOVA for continuous data

^b^SES based on tertiles from total monthly expenditures for Indian sites and total household asset score for Matlab, Bangladesh

**Table 2:** Prevalence of correlates of recommended physical activity status (RPALs) in rural South Asian children aged 5–14 years

Percentages (row)	Active mode of travel to school (*n* = 375)	Leisure-time physical activity ≥1 h per day (*n* = 393)	Sedentary activity ≤2 h per day (*n* = 308)
Age group			
5–9 years	57.5	79.8	62.1
10–14 years	74.2	70.4	50.5
Sex			
Boys	61.7	80.0	59.7
Girls	72.1	66.2	51.5
Site			
Matlab	62.0	80.2	37.0
Goa	69.4	78.8	67.3
Chennai	69.2	59.6	64.8
Highest household education status			
0–5 years of education	65.6	76.0	61.3
6 to 12 years of education	68.9	76.3	54.8
Graduate/Post graduate	59.7	63.2	45.8
Socio-economic status^a^ (SES)			
Low	69.2	70.3	56.3
Medium	64.7	75.3	57.6
High	69.1	75.6	53.1
Leisure-time physical activity (at school + at home)			
< 1 h/day	67.2	NA	53.8
≥ 1 h/day	65.1		55.1
Sedentary activity			
≤ 2 h/day	67.5	75.2	NA
> 2 h/day	64.7	74.2	
Mode of transport to school			
Active transport (walk/bicycle)	NA	73.9	56.8
Motorized transport		75.7	53.8
Fruit vegetable intake			
< 5servings/day	67.2	74.4	58.8
≥ 5servings/day	63.2	76.1	35.3
Processed food intake			
< 1serving/day	67.6	71.6	59.5
≥ 1serving/day	64.5	81.7	46.2

^a^SES based on tertiles from total monthly expenditures for Indian sites and total household asset score for Matlab, Bangladesh

*NA* Not Applicable

**Table 3:** Multivariate adjusted correlates of recommended physical activity status in rural South Asian children aged 5–14 years

	Odds Ratio (95 % CI) (*p*-value)		
	Active mode of travel to school	Leisure-time physical activity ≥1 h per day	Sedentary activity ≤2 h per day
Age group			
5–9 years	1.0 (Ref)	1.0 (Ref)	1.0 (Ref)
10–14 years	2.0 (1.3, 3.0) (*p* = 0.001)	0.7(0.4, 1.2) (*p* = 0.30)	0.6(0.4, 0.9) (*p* = 0.02)
Sex			
Boys	1.0 (Ref)	1.0 (Ref)	1.0 (Ref)
Girls	1.7 (1.1, 2.5) (*p* = 0.01)	0.4(0.2, 0.6) (*p* < 0.0001)	0.7(0.4, 1.1) (*p* = 0.14)
Site			
Matlab	1.0 (Ref)	1.0 (Ref)	1.0 (Ref)
Goa	1.2 (0.7, 2.1) (*p* = 0.43)	0.9(0.4, 1.7) (*p* = 0.80)	3.5(2.1, 6.1) (*p* < 0.0001)
Chennai	1.1 (0.6, 1.9) (*p* = 0.71)	0.3(0.1, 0.6) (*p* < 0.0001)	2.5(1.5, 4.3) (*p* = 0.001)
Highest household education status			
0–5 years of education	1.0 (Ref)	1.0 (Ref)	1.0 (Ref)
6 to 12 years of education	1.3 (0.8, 2.1) (*p* = 0.24)	1.2(0.6, 1.9) (*p* = 0.58)	0.6(0.3, 0.9) (*p* = 0.03)
Graduate/Post graduate	0.8 (0.4, 1.6) (*p* = 0.59)	0.4(0.2, 0.9) (*p* = 0.03)	0.5(0.2, 1.0) (*p* = 0.07)
Socio-economic status^a^ (SES)			
Low	1.0 (Ref)	1.0 (Ref)	1.0 (Ref)
Medium	0.7 (0.4, 1.2) (*p* = 0.21)	1.1(0.6, 2.0) (*p* = 0.59)	0.9(0.5, 1.6) (*p* = 0.90)
High	0.9 (0.5, 1.6) (*p* = 0.87)	1.0(0.5, 2.0) (*p* = 0.78)	0.6(0.3, 1.1) (*p* = 0.17)
Leisure-time physical activity (at school + at home)			
< 1 h/day	1.0 (Ref)	NA	1.0 (Ref)
≥ 1 h/day	1.0 (0.6, 1.7) (*p* = 0.85)		0.9(0.6, 1.5) (*p* = 0.9)
Sedentary activity			
≤ 2 h/day	1.0 (Ref)	1.0 (Ref)	NA
> 2 h/day	1.2 (0.8, 1.8) (*p* = 0.34)	0.9(0.6, 1.6) (*p* = 0.97)	
Mode of transport to school			
Active transport (walk/bicycle)	NA	1.0 (Ref)	1.0 (Ref)
Motorized transport		1.1(0.6, 1.7) (*p* = 0.82)	1.2(0.8, 1.8) (*p* = 0.32)
Fruit vegetable intake			
< 5servings/day	1.0 (Ref)	1.0 (Ref)	1.0 (Ref)
≥ 5servings/day	1.3 (0.7, 2.6) (*p* = 0.35)	0.7(0.3, 1.4) (*p* = 0.29)	0.5(0.2, 1.0) (*p* = 0.07)
Processed food intake			
< 1serving/day	1.0 (Ref)	1.0 (Ref)	1.0 (Ref)
≥ 1serving/day	1.0 (0.6, 1.7)(*p* = 0.82)	2.3(1.2, 4.1) (*p* = 0.006)	0.9(0.5, 1.4) (*p* = 0.73)

^a^SES based on tertiles from total monthly expenditures for Indian sites and total household asset score for Matlab, Bangladesh; *NA* Not Applicable

### Correlates of achieving recommended levels of physical activity

The correlates of recommended physical activity levels (RPALs) in rural children aged 5-14 yrs are presented in Tables 2 and 3 as proportions and multivariate adjusted odds ratios respectively.

#### Mode of travel to school

Older children aged 10–14 years (OR = 2.0; 95 % CI: 1.3, 3.0) and girls (OR = 1.7; 95 % CI: 1.1, 2.5) were more likely to use active forms of travel to school than younger children and boys respectively. There was no association between other socio-demographic (household education and SES status), diet (fruits-vegetables and processed food) and other forms of physical activity (leisure-time activity and sedentary activity).

#### Leisure-time physical activity

Boys (OR = 2.5; 95 % CI: 1.5, 4.0) and children living in rural Bangladesh (OR = 3.0; 95 % CI: 1.6, 5.5) reported more leisure-time physical activity than girls and children of other sites respectively. Leisure-time physical activity was lower in children coming from households with higher education status (i.e., at least graduation) (OR = 0.4; 95 % CI: 0.2, 0.8). Children who reported higher levels of leisure-time physical activity were more likely to consume processed food (≥1 serving per day; OR = 2.3; 95 % CI: 1.2 to 4.1). There was no association observed for leisure-time physical activity with SES based on total monthly expenditure or asset, fruits-vegetables consumption or other forms of physical activity (mode of travel to school and sedentary activity).

#### Sedentary activity

Younger children (5–9 years) had less sedentary activity outside school hours than older children (OR = 1.6; 95 % CI: 1.07, 2.4), or living in Goa (OR = 3.5; 95 % CI: 2.1, 6.1) or Chennai (OR = 2.5; 95 % CI: 1.5, 4.3). Children residing in households with lower education status (up to 5 years of education) were less sedentary (OR = 1.6; 95 % CI: 1.02, 2.7). There was no association found for sedentary activity with diet (fruits-vegetables and processed food) and other forms of physical activity (mode of travel to school and leisure-time physical activity).

#### Associations between physical activity levels and continuous outcome measures (BMI-z- score and waist- z-score)

In multi-variate analyses, sedentary activity ≤2 h per day was associated with significantly lower BMI (β-coefficient (95 % CI) = −0.3(−0.5, −0.08), *p* = 0.008) and waist-z-scores (β-coefficient (95 % CI) = −1.1(−2.2, −0.07), *p* = 0.03) after adjusted for site, SES, highest household education, processed food and fruit-vegetable consumption (Tables 4 and 5). There were no significant associations between leisure-time physical activity or active travel to school and measures of adiposity.

**Table 4:** Associations between physical activity levels and BMI-z-scores in rural South Asian children aged 5–14 years

Type of physical activity	Age and sex adjusted^a^ β-coefficient (95 % CI)	Multivariate-adjusted^b^ β-coefficient (95 % CI)
Mode of travel		
Motorized transport	0.00 (ref)	0.00 (ref)
Active transport	−0.1 (−0.3, 0.05)	−0.1 (−0.3, 0.1)
*p*-value	0.14	0.28
Leisure-time physical activity		
< 1 h/day	0.00 (ref)	0.00 (ref)
≥ 1 h/day	−0.06 (−0.3, 0.16)	−0.09 (−0.3, 0.16)
*p*-value	0.56	0.47
Sedentary activity		
> 2 h/day	0.00 (ref)	0.00 (ref)
≤ 2 h/day	−0. 2 (−0.4, − 0.04)	−0.3 (−0.5, −0.08)
*p*-value	0.01	0.008

^a^The outcome BMI-z- score is adjusted for age and sex

^b^Adjusted for site, SES (tertiles), Highest household education in categories, processed food consumption as binary, fruit-vegetable consumption as binary, leisure-time physical activity as binary (for mode of travel and sedentary activity), active mode of travel as binary (for leisure-time physical activity and sedentary activity) and sedentary activity as binary (for leisure-time physical activity and active mode of travel)

**Table 5:** Associations between physical activity levels and waist z-scores in rural South Asian children aged 5–14 years

Type of physical activity	Age and sex adjusted^a^ β-coefficient (95 % CI)	Multivariate-adjusted^b^ β-coefficient (95 % CI)
Mode of travel		
Motorized transport	0.00 (ref)	0.00 (ref)
Active transport	−0.7 (−1.6, 0.2)	−0.4 (−1.5, 0.6)
*p*-value	0.15	0.37
Leisure-time physical activity		
< 1 h/day	0.00 (ref)	0.00 (ref)
≥ 1 h/day	0.2 (−0.8, 1.2)	−0.1 (−1.2, 1.1)
*p*-value	0.70	0.86
Sedentary activity		
> 2 h/day	0.00 (ref)	0.00 (ref)
≤ 2 h/day	−0.5 (−1.4, 0.4)	−1.1 (−2.2, −0.07)
*p*-value	0.28	0.03

^a^The outcome waist-z- score is adjusted for age and sex

^b^Adjusted for site, SES (tertiles), Highest household education in categories, processed food consumption as binary, fruit-vegetable consumption as binary, leisure-time physical activity as binary (for mode of travel and sedentary activity), active mode of travel as binary (for leisure-time physical activity and sedentary activity) and sedentary activity as binary (for leisure-time physical activity and active mode of travel)

## Discussion

We report the patterns and correlates of recommended physical activity levels (RPALs) in rural South Asian children. Two thirds (66.7 %) used active mode of travel to school, three quarters (74.6 %) had ≥ 1 h/day of leisure-time physical activity and 55.7 % had ≤2 h of sedentary activity every day. Older (10 to 14 years of age) and female children travelled actively to school but girls participated in less leisure-time physical activity after school hours (median (IQR):77.1 (45.7, 105.7) min/day vs. 94.6 (70.0, 137.1) min/day in boys; *p* < 0.0001). Although children living in rural Matlab (Bangladesh) reported more leisure-time physical activity daily than children in rural Chennai (India; median (IQR): 94.2 (68.5,140) min/day vs. 68.5 (45.7,89.6) min/day; *p* < 0.0001), they also reported more sedentary activity outside school hours (median (IQR): 180 (90,240) min/day vs. 90 (60,125.8) min/day in Goa vs. 120 (72.5,150) min/day in Chennai). Children who belonged to households of higher education (secondary level or higher) were more sedentary and independently reported less leisure-time physical activity. Overall, only 25.2 % of children met the RPALs for all three categories i.e., active travel to school (any), ≥ 1 h/day leisure-time physical activity and ≤2 h sedentary activity every day.

We are not aware of similar data on rural children in South Asia for comparison. However, with reference to a school-based study from Wardha, Maharashtra, India the prevalence of physical inactivity was high (57.9 %) among children and adolescents aged 6 to 18 years [35]. In this study, although they used WHO - Global School-based Health Survey questionnaire, a modified physical activity score was calculated for each child that was adapted from a scoring system for adults. This could have been the reason for a high prevalence of physical inactivity reported in the study compared to our study. Studies in urban India and Bangladesh have consistently reported higher prevalence of physical inactivity in children and adolescents than levels documented in our study [1, 9, 10]. However, these studies suggest that physical inactivity is more common among girls and older children (22.2–61.9 % in girls vs. 18.3–47.4 % in boys) [1, 10] similar to our study. Studies on South Asian children (Indians and Bangladeshis) in UK found significantly lower levels of physical activity in South Asian children compared to European children with Bangladeshi children as the most sedentary amongst South Asians [36]. Some studies of UK primary school children found no differences between overall levels of physical activity and mode of transport to school [37, 38] similar to that found in our study, but the results are to be interpreted with caution and cannot be directly applied to children living in rural south Asia.

We found that less sedentary activity (≤2 h per day) was associated with significantly lower BMI- and waist-z-scores across all sites. Although not significant we found a weak inverse association of active mode of travel to school and greater leisure-time physical activity (≥1 h per day) with BMI-z-scores and waist-z-scores across all sites. These findings may reflect that independent of other forms of physical activity, sedentary behaviour is associated with various unfavourable physiological outcomes including weight related problems [1–3, 7] as well as the very low prevalence of overweight in our sample. Although the prevalence of over-weight or obesity in rural children aged 6–18 years has been reported at 2.2–9.7 % from two populations of India [39, 40], the association of physical activity patterns and weight outcomes is rarely reported from these rural regions [35]. In our rural children we found TV watching (49.3 %) as the main sedentary activity followed by reading (43.9 %), which is similar (i.e., long TV viewing) to what is reported in urban children and adolescents of India and Bangladesh [3, 11–18]. Although significant associations of weight-related outcomes and physical activity have been reported in urban children, similar data is distinctly lacking among rural South Asian children.

### Strengths/limitations of study

To our knowledge, these results are the first to report prevalence and correlates of recommended physical activity levels (RPALs) in rural south Asian children. In view of the lack of data from LMICs on rural children, this study is important to inform future monitoring and emerging policies in this area. A major limitation of our study is its cross-sectional nature, which prevents any causal inferences about the associations being observed. Additional limitations include the lack of a gold standard to validate self-reported levels, potential misclassification of physical activity levels and the potential for residual confounding. Another limitation is that the questionnaire grouped public transport (bus) with other ‘inactive’ modes of motorised transport such as cars and mopeds. Public transport is considered an active mode of travel as it generally involves walking or cycling to transport access points or interchanges [32]. Although we used a validated PA questionnaire, details on chores or activities that children are involved in, which are related to physical activity outside of school may have been missed out. We addressed this aspect in the questionnaire but the information collected reflects more on sports or games rather than chores or household or other work. Moreover, the questionnaire did not allow us for the calculation of metabolic equivalents of activity. These could have reflected in our results.

### Implications for policy/practice

Schools and communities have been identified as important settings for health promotion activities and interventions for children [41]. Thus interventions at both community and school levels are required for adequate policy actions regarding physical activity in children [28]. Further potential concerns with respect to physical activity and policies for South Asian countries include road safety and air pollution in terms of active transport to school, lack of space and child safety. Changing patterns of physical activity due to academic pressures in terms of leisure-time physical activity and lack of awareness regarding sedentary activity should also be considered. Maintaining the relatively high levels of active travel identified in this study through investment in public transport, cycling and walking infrastructure is important [34]. Ongoing monitoring and longitudinal studies of physical activity in rural south Asian children are important to better characterize trends over time and behavioural, social and environmental factors influencing PA in rural settings.

## Conclusion

Only one quarter of children achieved recommended physical activity levels in all three categories of physical activity (i.e., active travel to school, ≥ 1 h/day leisure-time physical activity and ≤2 h sedentary activity every day) in rural South Asia. Age, gender, household education, and geographical location were associated with RPALs. Less sedentary activity (≤2 h per day) may have beneficial effects on adiposity. These findings may indicate that low levels of physical activity and associated risks of overweight and obesity may no longer be restricted to children living in urban, affluent communities in South Asia. Improved understanding of physical activity levels in rural children is important given the rapid social and economic changes occurring in the region.

## Abbreviations

BMI, body mass index; CDRF, chronic disease risk factor study; LMICs, low-middle income countries; NCD, non-communicable Disease; PA, physical activity; RPAL, recommended physical activity levels; SANCD, South Asia network of chronic disease; SD, standard deviation; SES, socio-economic status; WHO, World Health Organization
